# Complete Genome Sequence of *Mycoplasma bovigenitalium* Strain HAZ 596 from a Bovine Vagina in Japan

**DOI:** 10.1128/genomeA.01554-16

**Published:** 2017-02-09

**Authors:** Eiji Hata, Kazuya Nagai, Kenji Murakami

**Affiliations:** a Dairy Hygiene Unit, Division of Pathology and Pathophysiology, Hokkaido Research Station, National Institute of Animal Health (NIAH), National Agriculture and Food Research Organization (NARO), Toyohira-ku, Sapporo, Hokkaido, Japan; b Department of Veterinary Medicine, Faculty of Agriculture, Iwate University, Morioka, Iwate, Japan

## Abstract

*Mycoplasma bovigenitalium*, a mycoplasmal species involved in various bovine diseases, including genital disease and mastitis, is also a commensal microorganism that inhabits the bovine genital organs. We present here the complete 853,553-bp genome sequence of *M. bovigenitalium* strain HAZ 596, which was isolated from a bovine vagina in Japan.

## GENOME ANNOUNCEMENT

*Mycoplasma bovigenitalium* causes various bovine illnesses, including genital disease and mastitis, and is a commensal microorganism that inhabits the bovine genital organs (1–3). Mycoplasmal bovine mastitis often leads to huge economic losses, and *M. bovigenitalium* is isolated from approximately 10% of milk from dairy herds (4). Although a draft genome sequence of *M. bovigenitalium* was previously reported (5), to the best of our knowledge, its complete genome sequence has not yet been reported. We present here the whole-genome sequence of strain HAZ 596, which was isolated in 1993 from the vagina of a mastitic cow in Japan.

Total genomic DNA was prepared from *M. bovigenitalium* strain HAZ 596 and subjected to 454 titanium sequencing at Iwate University, Morioka, Japan. The resulting reads were assembled *de novo* using GS De Novo Assembler software version 2.7 (Roche), yielding 50 contigs with 102.1× coverage. An analysis of the contig ends together with PCR amplification and amplicon cloning showed that the 853,553-bp genome had a closed-ring structure. After performing an initial automated annotation using Microbial Genome Annotation Pipeline version 2.20 at the DNA Data Bank of Japan (http://migap.ddbj.nig.ac.jp/mgap/jsp/index.jsp) (6–8), we carried out manual curation, followed by verification of potential pseudogenes by PCR and Sanger sequencing. We confirmed 624 open reading frames, 24 pseudogenes, 31 tRNAs, and two sets of each rRNA (5S, 16S, and 23S rRNA) in this genome sequence. The guanine-cytosine (GC) content was 29.10%.

The amino acid sequences of most genes containing rRNA genes in* M. bovigenitalium* HAZ 596 exhibited high similarity to those of the genes encoded by *Mycoplasma simbae* and *Mycoplasma californicum* (9). Moreover, a total of 21 genes containing pseudogenes were estimated to be mobilomes, and 16 were transposases. The amino acid sequences of the transposase genes showed high similarity to those of the transposase genes encoded by *Mycoplasma dispar*, *M. californicum*, and *Mycoplasma conjunctivae*.

In this genome, Bertin et al. (10) confirmed the genes of proteins involved in the synthesis of capsular polysaccharides (galactan), which have been suggested to be important mycoplasmal etiologic agents, i.e., phosphotransferase system IIB components (MBVG596_0167), phosphomannomutase (MBVG596_0220 and MBVG596_0748), UTP-glucose-1-phosphate uridyltransferase (MBVG596_0542), UDP-glucose 4-epimerase (MBVG596_0050), UDP-galactopyranose mutase (MBVG596_0051), BcsA glycosyltransferases (MBVG596_0841, MBVG596_1060, and MBVG596_1132), and WcaA glycosyltransferases (MBVG596_0016, MBVG596_0750, and MBVG596_1133). With respect to the genes of proteins involved in the production of reactive oxygen species (ROS), which have been suggested to be important mycoplasmal etiologic agents (11), the genes of proteins involved in the membrane-located ATP-binding cassette transporter system, i.e., glycerol ABC transporter ATP-binding protein (MBVG596_0355, MBVG596_0445, and MBVG596_0628) and glycerol ABC transporter permease (MBVG596_0357, MBVG596_0359, MBVG596_0443, and MBVG596_0444), have been confirmed in this genome. However, the present strain may not produce ROS, because no glycerol-3-phosphate oxidase gene was encoded in this genome (11).

The genomic sequence of *M. bovigenitalium* will provide a foundation for future research on this species. Ultimately, it is hoped that the present study will contribute to the reduction of mycoplasmal bovine diseases.

### Accession number(s).

The whole-genome sequence has been registered at DDBJ/EMBL/GenBank under GenBank accession no. AP017902.
